# Philadelphia Hospital

**Published:** 1839-02-16

**Authors:** 


### CLINICAL LECTURES.

### PHILADELPHIA HOSPITAL.

### ON PNEUMONIA—(CONTINUED.)

*Saturday, January 12th.*—Dr. Gerhard remarked:

If the general symptoms of pneumonia are uncertain, we are happily enabled to recognise the disease with great certainty by means of the physical signs. Nay, we may go much further, and can point out the exact portion of the lung which is affected—the stage of the disease—and the rapidity with which it advances or declines. The physical signs do not even stop here; we can appreciate, by their aid, the alterations left in the lung after the disease has completely ceased, and are able to ascertain, in a positive way, whether the disease has left behind any permanent traces of its existence.

In order to understand clearly the physical signs of pneumonia, it is necessary for us to examine the changes which occur in the anatomical structure of the lung in every stage of the disease. This we can do very readily, by recollecting the appearances which I demonstrated to you a short time since, in the lungs of a patient dead of pneumonia, complicated with arachnitis. You then saw, as is most frequently the case, the different stages of the disease exemplified at one and the same time. At the upper portion of the lung, the tissue was injected with various shades of red; varying from a bright rose tint to a deep purple, and containing a much larger quantity of blood than usual, which exuded upon slight pressure. The tissue was somewhat friable, but contained air in every portion of its extent. The shades of redness are so various that you may, if you choose, subdivide the first stage of pneumonia into two or three varieties; but as little practical advantage results from these minute distinctions, it will be better for you to regard all these various phenomena as an indication of shades of the same pathological condition. The bronchial tubes are reddened in this stage, and slightly thickened, containing a little thin mucus. It is extremely difficult to distinguish this variety of the disease from simple congestion of the lung. Indeed, it is not possible always to do so. But in most cases of true pneumonia, the difficulty is unimportant, for portions of the lung are almost always inflamed to a greater degree, and advanced at the second or third stage.

The second stage of pneumonia is much better characterized. In this stage the blood which accumulates in the lung, in consequence of inflammation, becomes, as it were, incorporated with its tissue, and gives an unusual degree of density to it. The lung sinks in water, contains no air, has lost its elasticity, and is friable upon pressure, which forces from it a reddish liquid. The vesicles of the lung distended with coagulated blood, or perhaps with lymph, are turgid, and swell out when the lung is torn, giving to it a peculiar granulated appearance. The bronchial tubes are now much inflamed, their mucous membrane, exteriorly, red, thickened, and covered with an opaque, thick, whitish, and tenacious fluid, very similar to the matter expectorated—that is, the larger tubes, for the smaller ones are gradually compressed by the progressing of the second stage. Their sides are pressed together by the distended lung, and air no longer enters them. The second stage is the most characteristic of pneumonia, both from its physical and general signs.

The third stage presents a change in colour; the tissue is at first marbled with yellow, which contrasts with the red colour of the second stage. Gradually the yellow colour extends, until the whole inflamed portion is of a yellow or greenish tint; the vesicles are less distended, and the granulated appearance is less evident, while the bronchial tubes are still greatly inflamed, and become totally impervious to the air. The colour results from the infiltration of pus, which gradually extends through the lung; in proportion as the infiltration becomes complete, the tissue of the lung loses its consistence, and in extreme cases may be washed away by directing a small stream of water upon it, leaving the bronchial tubes and vessels without the intervening vesicular structure. In most cases, however, the softening is less complete; the lung is readily broken down by pressure, into a yellowish pulp, but its texture is still preserved. In most cases which terminate fatally, a portion of the lung, at least, has passed into the third stage.

A fourth period may occasionally be observed in pneumonia; that is, when the pus is no longer infiltrated with the cellular tissue of the lung, but is collected into well-defined abscesses. These are formed very slowly in pneumonia; it would seem that the bronchial tubes and vessels, which are disseminated so numerously throughout the tissue of the lungs, prevent, in a great measure, the collection of the pus into masses. When an abscess takes place, it presents very nearly the same characters as are observed in any other portion of the cellular tissue of the body, and it is surrounded by the usual false membrane. These abscesses are very rarely found in the dead body, because those patients who have passed through the early stages of the disease almost uniformly recover—the formation of pus being, in fact, the best evidence possible of the termination of the diseased action. When abscesses are cured, in some cases, entire adhesion takes place between their walls, and in others the cavity becomes fistulous, and is lined with a firm, but transparent membrane, which secretes merely a small quantity of mucus.

The pathological changes occurring in cases of pneumonia, which have not advanced as far as the second stage, are also worthy of note. In cases which have been cured while still in the first stage, or even in the beginning of the second stage, the lung gradually resumes its former appearance, without leaving any trace of the inflammation. In other words, the resolution is complete. When the disease has advanced to the third stage, or when the second stage has existed for several days, the lung is slow to recover its normal condition; it remains for a long time flaccid, and contains very little air,—in some cases it is even contracted by adhesions which confine it closely to the ribs. When you recollect that the lung does not immediately return to its natural state, you can readily account for the permanence of the physical signs of the disease, which always persist after the general symptoms have more or less completely disappeared.

In describing to you the pathological appearances observed in pneumonia, I am merely recapitulating what I have already demonstrated to you in the earlier part of the course,—and you should recall to your recollection the aspect and physical condition of the lungs in each stage of the disease, if you desire to form to yourselves a clear conception of the changes in the physical signs of disease which correspond with them.

The physical signs are very regular in their developement, and are, to a certain extent, illustrated by the case of pneumonia which I described as an example of the simplest form of the disease. In the first stage you will observe, in a considerable number of patients, a decided crepitant rhonchus; perhaps the greater number of patients to whom you are called will offer this phenomenon at your first examination. Still, you should not conclude that this sign is the first physical evidence of pneumonia. I have long since satisfied myself, that in by far the greater number of cases, both in children and in adults, the earliest signs of pneumonia are not the crepitant rhonchus. The disease generally begins about the root of the lung, at a certain distance from the surface, and the signs on auscultation depend upon two different circumstances—upon the obstruction to the passage of the air through the engorged portion, and the increased rapidity with which it is driven through that part which is still quite permeable. Hence you will find that the respiration is more or less rude or rough, and at the same time imperfectly vesicular in the part of the lung which is the seat of the inflammation. At the same time we often detect a loud, puerile respiration, surrounding, as it were, the inflamed portion, and more or less intermixed with the rude respiration. These modifications of the respiratory sound occur very early in the disease, and are often accompanied by the more doubtful functional signs which indicate the beginning of pneumonia.

The crepitous rhonchus is rarely developed in its greatest perfection until a portion of the lung has passed into the second stage. You then discover the bronchial respiration around the large tubes, and the crepitant rhonchus near the part of the chest in which it is heard. If a strong inspiration be taken, the crepitant rhonchus is quickly reproduced in the parts where the bronchial respiration is alone heard when the inspiration is but moderately rapid. You have all heard these sounds in several cases of pneumonia, which have been recently in the hospital; and you must have remarked in one of the last of these cases, that the crepitant rhonchus which had entirely ceased near the summit of the lung, was immediately reproduced by directing the patient to cough with some force,—a strong inspiration afterwards forced the air into the bronchial tubes, which had been, in great part, freed from the accumulation of mucus by the cough, and a very distinct crepitus followed. In the case which formed the subject of the last lecture, the crepitus was loud, and well-marked in the whole extent of the inflammation, which proved that the disease had not passed so far through the second stage as to prevent the air entirely from entering the tissue of the lungs.

The cause of the crepitant rhonchus is probably twofold; it results in part from the viscid mucus, which obstructs the smallest tubes, and breaks up with a crackling noise when the air is forced into them, and in part from the rigidity of the walls of the vesicles, which give rise to the peculiar sound when the air distends them. This supposition is not merely theoretical, but is founded upon observation.

I have already stated that bronchial respiration was almost always combined with the crepitous rhonchus. It is not necessary for me to describe minutely the characters of this sound, which, as you know, consists in a blowing sound heard both in the inspiration and expiration. At times, it is so loud and distinct, as to convey to the observer an impression that the air is actually blown into his ear; in this case, it is called tubal respiration. What I wish to impress upon you is, that this sound is dependent upon the anatomical structure of the lung, as well as upon its induration; hence, wherever you find that the bronchial tubes are very large, and of course give free passage to the air, you will remark that the bronchial respiration is unusally distinct. It is, therefore, most evident at the root of the lungs; that is, at their posterior margin, near the place of union of the upper and lower lobes, precisely at the point where pneumonia most frequently occurs, and is most rapid in its progress. Next to the root of the lungs, you will find that the summit of the right lung is most favourable for its production, and afterwards the summit of the left. The inferior portion of the lungs is much less adapted for the promotion of bronchial respiration, because the tubes are much smaller, and, from their direction, are very easily obstructed by thick mucus, which accumulates in them, at the same time that they are compressed by the distension of the surrounding pulmonary tissue. Hence the bronchial respiration ceases very early in most cases of pneumonia, throughout the lower portion of the lungs. The only sign which is then perceptible, is perhaps a slight crepitus, and an absence of vesicular sound. You are aware that bronchophony is always proportioned to the loudness of the bronchial respiration, and is produced by the same physical state of the lungs.

I have but a word or two to say respecting the physical signs of the third stage. The tissue of the lung remains impervious to the air; hence you find that the percussion remains as dull as it was throughout the whole of the second stage. The bronchial respiration ceases, or becomes excessively feeble; while every powerful inspiration, or even a slight cough, will give rise to a loose mucous rhonchus, from the quantity of purulent liquor contained in the larger bronchial tubes. Bronchophony diminishes in the same proportion as the bronchial respiration.

When the disease declines, after having reached the third stage, the mucous rhonchus gradually ceases, and the vesicular murmur returns. If it has not advanced farther than the second stage, the bronchial respiration is gradually replaced by a loose, crepitant rhonchus, which subsides as the vesicular murmur returns. When the hepatization has been very complete, the respiration does not resume its natural character immediately after the disease is cured, but remains for a time rough, and the vesicular murmur is in a great degree lost. The slow return of the vesicular murmur is readily explained by the indurated condition of the lung, which is so constantly observed in patients who have died of acute diseases soon after recovering from pneumonia.

These details seem necessary to enable you to understand this disease in its more complicated state. You have learned from the demonstrations of the physical signs which I have made to you in the living patient, as well as from the examination of the lungs in cases which proved fatal, that the phenomena are very regular, and present extremely few exceptions. We are accustomed, therefore, to allude very frequently to pneumonia, as one of the best illustrations of the truth of the physical signs, and one of the most useful objects of study, for those who are desirous of becoming acquainted with these signs; and although much of what I have detailed to you will be trite and uninteresting to those who are already familiar with the subject, I should not have the same assurance that I should be understood in our study of the subject, if I had omitted this grouping of the signs and pathological lesions of pneumonia. After you have acquired a knowledge of the disease in its simple form, you will find the study of its difficult complications greatly facilitated.

---

### DISEASES OF THE EAR.

*Saturday, February 2d.*—Dr. Horner, whose tour of service commenced this day in conjunction with Dr. Edward Peace, entered the amphitheatre at ten o'clock, and remarked:—

Gentlemen,—The term of my colleague, Dr. Gibson, having just expired, the duty of continuing the surgical clinic in this house has devolved upon me. A very full occupation of my time by my obligations as a lecturer in the University, and other engagements, make any further appropriation of it scarcely desirable. I may also add that the ability displayed by my predecessor, and his very evident familiarity with the duties of this station, render it difficult for one who has so seldom officiated as a clinical lecturer, and never regularly so, to present favourably his services. I have repeatedly witnessed with satisfaction and improvement the very excellent discipline introduced into the clinical course of this house, both in surgery and in medicine, offering as it does the amplest opportunities of acquiring practical knowledge in both these branches. Had I been governed merely by private considerations of convenience and propriety, I should have declined the task assigned me. But a deference to the expressed opinions of my colleagues in this institution, and a desire on my part to sustain as far as was in my power its interests and its usefulness, were paramount considerations. I felt, in fact, zealous for an institution which for its convenience and extent is unrivalled in our country; the administration of which is excellent; and which presents the solitary objection of being rather farther off from our houses than we could wish, and consequently requiring more time and a greater sacrifice of comfort in attending it than we would desire, were the liberty of choice still left.

The very thorough exposition of the existing surgical diseases of the house, and of such as have previously occurred during the season, by my predecessor, leaves but a limited choice of subjects, without the hazard of repetition. I therefore have thought that it would be beneficial to take up a group of affections of high interest, but which unfortunately have been very much neglected by the profession at large—I mean the diseases which assail the organ of hearing, and which are very properly classified under the term *otiatric surgery.* An additional incentive to their study is, the circumstance that such diseases being generally overlooked, I may indeed say almost repudiated, by regular practitioners, have to a great extent fallen under an ignorant empirical management. Even where better knowledge has existed, it has been connected with so much mystification, and such extraordinary claims to special skill, that it has hitherto been placed in an extremely unsatisfactory relation to the profession and to the public. I propose, therefore, to show you that there is no great mystery in treating diseases of the ear; that the rules are simple, the affections few; and that the general results of treatment are such as to encourage us to undertake them. In proof of this assertion I will here exhibit and explain a "Tabular view of the Curability and Frequency of Diseases of the Ear," by Dr. Kramer, a gentleman of whose labours in this department of our science I shall have occasion to speak more fully presently.

| NAME OF THE DISEASE. | Incurable and not treated. | Cured. | Relieved. | Uncured. | TOTAL. | | | |
|---|---|---|---|---|---|---|---|---|
| OF THE AURICLE. | | | | | | | | |
| Erysipelatous Inflammation . . . . . . . . . | .. | 1 | .. | .. | 1 | 3 | | |
| Scirrhous Degeneration . . . . . . . . . . | .. | 2 | .. | .. | 2 | | | |
| Furuncle . . . . . . . . . . . . . . . . | .. | .. | .. | .. | .. | | | |
| IN THE MEATUS EXTERNUS. | | | | | | | 85 | In the External Ear. |
| Erysipelatous Inflammation . . . . . . . . . | .. | 17 | .. | .. | 17 | 46 | | |
| Inflammation of the Glandular Integument . . . | 3 | 9 | 13 | .. | 25 | | | |
| Inflammation of the Cellular Tissue . . . . . | .. | 2 | .. | .. | 2 | | | |
| Inflammation of the Periosteum . . . . . . | 2 | .. | .. | .. | 2 | | | |
| OF THE MEMBRANA TYMPANI. | | | | | | | | |
| Acute Inflammation . . . . . . . . . . . | .. | 1 | .. | .. | 1 | 36 | | |
| Chronic Inflammation . . . . . . . . . . . | 11 | 7 | 17 | .. | 35 | | | |
| IN THE CAVITY OF THE TYMPANUM AND EUSTACHIAN TUBE. | | | | | | | | |
| Inflammation of the Mucous Membrane, with Obstruction . . . . . . . . . . . . . . . | .. | 28 | 6 | .. | 34 | 55 | 55 | In the Middle Ear. |
| Inflammation of the Mucous Membrane, with Stricture of the Eustachian Tube . . . . . . . . . | 16 | .. | 3 | .. | 19 | | | |
| Inflammation of the Mucous Membrane, with Obliteration of the Eustachian Tube . . . . . . . | 1 | .. | .. | .. | 1 | | | |
| Inflammation of the Cellular Tissue of the Cavity of the Tympanum . . . . . . . . . . . . | .. | .. | .. | 1 | 1 | | | |
| IN THE LABYRINTH. | | | | | | | | |
| Erethitic Nervous Deafness . . . . . . . . . | 60 | 21 | 52 | 7 | 140 | 152 | 152 | In the Internal Ear. |
| Torpid Nervous Deafness . . . . . . . . . | 3 | 8 | 1 | .. | 12 | | | |
| Deafness and Dumbness . . . . . . . . . . | 8 | .. | .. | .. | 8 | | 8 | |
| | 104 | 96 | 92 | 8 | 300 | | 300 | |
| | | 188 relieved. | | | | | | |

Much of the professional reluctance to meddle with diseases of the ear has arisen, I am convinced, from a false view or estimate of its anatomy. We have been frightened by its intricacy. Of this intricacy there can be no doubt; but let us observe that it is one of mechanical arrangement, and not the complexity of a vast mass of materials diversely blended. Only a few materials enter into the composition of a house, and yet we may build a very simple or a very intricate house. But the study of such materials has no necessary connection with the intricacy of position; we may study them in their most simple or uncombined condition. In the same way the study of the ear is accomplished upon the basis of its elements or tissues, and not upon the basis of their position. If these elements, instead of a complex mechanical arrangement, were laid out like the squares of a checker-board, we should not hesitate to declare their diseases curable. With regard to the physiological action of remedies we have just the same opportunity in the body as if this were actually the case. It is only in the mechanical treatment that we are embarrassed, and a proper diligence in studying aural anatomy, and the requisite manipulations, will enable us to overcome many of these difficulties. Upon the principle just stated, the problem to be solved is simple enough; for the elementary tissues which enter into the composition of the ear are few in number, and of these a small portion only are commonly involved in disease. These elementary tissues are,—1. Bone. 2. Cartilage. 3. Skin. 4. Glandular structure. 5. Mucous membrane. 6. Condensed cellular tissue. 7. Serous membrane. 8. Blood-vessels and nerves. The muscular structure might be added, but it exists in such feeble proportion, that I do not think it worth while to bring it forward at present. The diseases of the ear fall almost exclusively, or in an overwhelming majority, upon four of these tissues—the dermoid, the glandular, the mucous, and the nervous, or the nervous and serous combined, for in fact it is impossible to separate them. We thus reduce the practical points of otiatric pathology to very few, and simplify, to an almost equal degree, the means of treatment. As to the hypothetical diseases, such as we might conceive a structure like the organ of hearing to be subject to, it would scarcely be reasonable to embarrass ourselves with the possibility of their being present, when there are so many probabilities, I might almost say, certainties that they are not.

Every affection of the ear must resolve itself either into a derangement of mechanism, or a derangement of tissue. The latter is most common. I shall now offer you an outline of these derangements in each division of the ear.

The mechanical derangements of the meatus externus arise from mechanical obstruction which is most commonly produced by an accumulation of ear wax, foreign bodies, or small tumours. These several conditions arrest audition simply by interposing a barrier to the vibrations of the air, in the same manner as we stop our ears with the finger.

In regard to the middle ear, its mechanical impediments to conducting a vibration of the air, may arise from a collection of mucus, or of any thing else which fills the tympanum, as blood or a tumour, or it may be caused by an obstruction of the Eustachian tube sensibly diminishing the vibrations of the air contained in the tympanal cavity, and consequently of the membrana tympani, in the same way as stopping up the hole in the side of a drum. Or, lastly, the vibrations of the membrana tympani may not be conveyed to the line of the ossicula auditus, owing to some interruption in the communication by fracture or dislocation of the latter; in which case the membrana tympani is probably only a barrier, and not an assistance in transmitting sound.

In regard to the labarynth or internal ear, we have no means of distinguishing its mechanical derangements. We might indeed imagine a great many, but they would be mere conjectures, more likely to lead us astray in the treatment of any one case than to establish rational signs.

The most general complaint with regard to the function of hearing, is its failing, or in other words, it is in a state of paracusis. There are examples also of hypercusis, or an extreme sensibility or exaltation of the function, making sounds incredibly distressingly sonorous. There are also sounds entirely deceptive, and from their not being real are called susurrus, or tinnitus, and they simulate all the noises under heaven. They may be considered as a sort of delirium of the sense, which, like that of the brain, sometimes renders it almost impossible to make a rational application of the sense in the midst of the agitations which are sure to visit it.

With a view of making more intelligible subsequent remarks, it may be as well to spend a few moments in studying the prominent traits in the anatomy of the ear.

[A demonstration was now made from some sections of the cranium prepared for the purpose.]

The early stage of acoustic medicine presents but little to admire or imitate. Its therapeutics consisted almost entirely of the application of stimulating articles to the meatus externus, such as garlic, onions, euphorbium, savine, hellebore, &c. &c. Most of the distinguished anatomical names of the fifteenth and sixteenth centuries, as Ingrassius, Eustachius, Fallopius, &c., are found in connection with efforts to advance this department of medicine. The result of their labours were some brilliant discoveries in the minute anatomy of the organ, but nothing useful, either therapeutically or pathologically was done. In 1724 Guyton, a postmaster at Versailles, more than two hundred years after the discovery of the Eustachian tube, by a lucky thought, injected his own Eustachian tube for the relief of deafness. This he did through the mouth. In 1741, Cleland, an Englishman, introduced the tube through the nose. Since then there have been successive stages of improvement, until the operation has become as settled, and almost as easy as catheterism of the bladder. There have been a vast number of writers in otiatric literature, but by most of them little has been contributed to the sciencc. To Itard, we are indebted for perfecting and developing the manner of injecting the middle ear through the Eustachian tube. To Deleau is due the merit of applying the air-donche for the same purpose. Otiatric surgery has received a new impulse within the last few years, by the publication of a treatise by Dr. Kramer, a German physician, who has made diseases of the ear an especial object of study, and by his clear and systematic arrangement, has contributed in a great measure, to remove the opprobrium which was before so justly attached to this important branch of surgery. I shall have frequent occasion to refer to Dr. Kramer, and shall adopt and follow his classification and views in many respects.

The deprivation of the function of audition is certainly one of the most serious calamities that can befall a person, involving as it does the mental and moral character of the individual. Those children who, from congenital malformation or from disease in very early life, are deprived of their hearing, are particularly objects of commiseration, owing to their social position.

A good deal of dispute has arisen as to the relative importance of particular portions of the ear. Itard has gone so far as to assert that the external ear is useless, and that no diminution of audition would take place from the destruction of the auricle, while, on the other hand, Buchanan attaches the greatest consideration to this constituent of the auditory apparatus. In order that the whole organ should be in the highest degree of perfection, a perfectly healthy condition of all its elements should exist.

It may not be improper here to call your attention to the subject of prophylaxis, or the adoption of such rules as will tend to preserve the integrity of the function of hearing. The agents which unquestionably exert the most injurious influence on the ear are *cold, and acute sounds.* Popular opinion endows cold with the property of invigorating the organ of hearing, which is a very erroneous and pernicious idea, for from its limited connection with the vascular system it is peculiarly unfitted to resist the effects of cold. While bathing, the ear should be protected by a plug of cotton in the meatus, and should be cleansed only with tepid water. As I have before stated that the most universal symptoms of aural disease was either an exaltation or a diminution of the functions of the ear, and that the latter was most frequent; in order to determine the precise extent of the morbid condition, a watch should be employed which admits of a definite and uniform standard of comparison. The human voice is too various and too liable to produce deceptive results to be relied on with any sort of confidence. Some complicated acoutemeters have been invented. but they are all inferior to the watch. From the particular organization of the ear, its affections are, for the most part, of a chronic character. According to Kramer not more than two out of an hundred cases are acute, or seen marked in the commencement with acute symptoms. There can be no doubt that some persons are particularly subject to aural maladies from hereditary predisposition, and advanced age brings additional liability to them, though the exposed and unprotected condition of this delicate organ is probably the most important predisposing cause. Among the exciting causes I have already mentioned cold, which undoubtedly is the first. Diseases of the ear are frequent sequelæ of the exanthemata, as scarlatina, measles, variola, &c., and of erysipelas. Also, the chronic cutaneous disorders, as crusta lactea, tinca capitis, &c. In scrofulous affections we continually see the ear involved, and in the Children's Asylum attached to this institution, where purulent ophthalmia is a very formidable affection, it is very apt to be complicated with catarrhal inflammation of the meatus auditorius.

The prognosis is by no means as bad as commonly anticipated, provided they receive early and continued attention. In 300 cases recorded promiscuously by Kramer, 188 were either completely relieved or materially benefitted; 104 were hopeless, and no attempt at relief was made; eight were totally unrelieved by any plan of treatment.

A careful local investigation is necessary in all cases at the outset. In proof of this, Kramer states that out of 300 patients he found the membrana tympani partially destroyed in 28, without its being suspected by the practitioners who previously had had charge of the cases, and who had been employing stimulating applications in their treatment.

I shall now proceed to exhibit to you such instruments as are commonly employed either in the investigation or treatment of diseases of the ear.

[The ear-syringe and speculum of Kramer, the lantern, and silver catheter for the Eustachian tube, the air-condenser, and ear-trumpets, were successively shown, and directions were given for their use, which was further practically demonstrated. In exhibiting the speculum, Dr. Horner dilated on the necessity of a careful local investigation of the meatus externus in diseases of the ear. He mentioned some cases related by Kramer, where ridiculous mistakes had occurred from the neglect of this precaution. He also related the case of a gentleman in his practice, who had been treated for vertigo, and apoplectic symptoms homœopathically. On an examination of the ear, a plug of wax half an inch long was found in each meatus. On their removal, all the disagreeable symptoms disappeared. The air-condenser used by Dr. Horner is of simple construction, and is adapted by himself. It consists of a simple air-tight tin cylinder, to which a syringe is fitted, and which forces in another atmosphere, the cock is then turned, and the flexible tube is adjusted. He has found it in all cases of sufficient power. The manner of testing by the air-donche and bougie, the permeability of the Eustachian tube were explained and demonstrated. A demonstration was also made of testing the permeability of the Eustachian tube by puncturing the membrana tympani. The meatus is corked up; by a tube previously fitted in the cork, air or water is injected into the middle-ear, which if the tube be pervious runs out through the nose.]

This operation, gentlemen, I have repeatedly performed, when from circumstances the patient wanted an immediate decision on his case; the knife with which I do it, is as you see, about a line in breadth and being introduced at the lower margin of the membrana tympani, a solitary puncture is made from the circumference to the centre. I have not known any inconvenience to arise from this trial, and the wound soon heals; more cases, however, are yet wanted to learn whether it is an operation which may be always done with impunity, and until this is settled I refain from recommending it as a general practice. It is a plan unquestionably preferable to that of Itard, who punctures the membrana tympani, and gropes about with the point of the syringe to find where the hole is.

[Three cases of catarrhal inflammation of the meatus externus in children were then brought forward, and their symptoms explained, and also the concomitance of this affection with the purulent ophthalmy, which the children of the asylum suffer so extensively with.

The lecture terminated by exhibiting several cases of frost bite, or congelation from cold, and directions for their treatment.]

