# Erratum in No. 7

**Published:** 1839-02-23

**Authors:** 


Erratum in No. 7.—In Prof. Horner's Clinical Lecture, page 110, fortieth line from top, first column, for "*making sounds incredibly distressingly sonorous,*" read "*making sounds inaudible to most persons audible, and the common sounds distressingly sonorous.*"

